# Label-free enumeration, collection and downstream cytological and cytogenetic analysis of circulating tumor cells

**DOI:** 10.1038/srep35474

**Published:** 2016-10-14

**Authors:** Manjima Dhar, Edward Pao, Corinne Renier, Derek E. Go, James Che, Rosita Montoya, Rachel Conrad, Melissa Matsumoto, Kyra Heirich, Melanie Triboulet, Jianyu Rao, Stefanie S. Jeffrey, Edward B. Garon, Jonathan Goldman, Nagesh P. Rao, Rajan Kulkarni, Elodie Sollier-Christen, Dino Di Carlo

**Affiliations:** 1 Department of Bioengineering, University of California, 420 Westwood Plaza, 5121 Engineering V, P.O. Box 951600, Los Angeles, CA 90095, USA; 2 Vortex Biosciences Inc., 1490 O’Brien Drive, Suite E, Menlo Park, CA 94025, USA; 3 Department of Surgery, Stanford University School of Medicine, MSLS Bldg, 1201 Welch Road, Stanford, CA 94305, USA; 4 Department of Pathology and Laboratory Medicine, University of California, Los Angeles, Los Angeles, CA 90095, USA; 5 Jonsson Comprehensive Cancer Center, Los Angeles, CA 90095, USA; 6 UCLA Santa Monica Hematology Oncology, 2020 Santa Monica Blvd, Suite 600, Santa Monica, CA 90404, USA; 7 California NanoSystems Institute, 570 Westwood Plaza, Building 114, Los Angeles, CA 90095, USA; 8 Division of Dermatology, UCLA Medical Center, 52-121 CHS, Los Angeles, CA 90095, USA.

## Abstract

Circulating tumor cells (CTCs) have a great potential as indicators of metastatic disease that may help physicians improve cancer prognostication, treatment and patient outcomes. Heterogeneous marker expression as well as the complexity of current antibody-based isolation and analysis systems highlights the need for alternative methods. In this work, we use a microfluidic Vortex device that can selectively isolate potential tumor cells from blood independent of cell surface expression. This system was adapted to interface with three protein-marker-free analysis techniques: (i) an in-flow automated image processing system to enumerate cells released, (ii) cytological analysis using Papanicolaou (Pap) staining and (iii) fluorescence *in situ* hybridization (FISH) targeting the *ALK* rearrangement. In-flow counting enables a rapid assessment of the cancer-associated large circulating cells in a sample within minutes to determine whether standard downstream assays such as cytological and cytogenetic analyses that are more time consuming and costly are warranted. Using our platform integrated with these workflows, we analyzed 32 non-small cell lung cancer (NSCLC) and 22 breast cancer patient samples, yielding 60 to 100% of the cancer patients with a cell count over the healthy threshold, depending on the detection method used: respectively 77.8% for automated, 60–100% for cytology, and 80% for immunostaining based enumeration.

Circulating tumor cells (CTCs) are believed to be responsible for cancer metastasis and represent potential biomarkers of disease progression^1^. Numerous studies have found a correlation between CTC presence/frequency and poor outcomes, indicating that the number of these cells are potential biomarkers of malignancy and can be used for diagnosis or prognosis^2^,^3^,^4^. Beyond enumeration alone, CTCs can provide a good source of malignant tissue for genetic analysis, protein expression analysis and drug testing, potentially leading to the development of non-invasive treatment monitoring and personalized therapies^5^,^6^. However, despite the interest in obtaining these cells, collection of CTCs is challenging because of their limited numbers (~1–500 CTCs per ml of blood), and presence among a very high number of contaminating white blood cells (WBCs) and red blood cells (RBCs).

To selectively separate these rare cells from blood, many “label-based” methods have been developed to isolate cells based on surface antigen-based capture (e.g. epithelial cell adhesion molecule, EpCAM)^7^,^8^,^9^, but these approaches are limited by heterogeneous inter/intra-tumor protein expression^10^,^11^. Addition of cocktails of antibodies that target a range of antigens partly addresses this issue^12^, but can lead to more off-target capture and reduced purity. Such techniques also remain expensive and labor-intensive. To circumvent some of these issues, negative selection (depletion) of the contaminating blood cells instead has been proposed^6^,^13^,^14^. This negative selection approach has the advantage of un-biased capture but achieves limited capture purity. Biophysical methods that leverage differences between CTC and blood cell sizes have been developed to circumvent this reliance on protein expression and provide a more cost-effective alternative^15^. These approaches include microfiltration either with paper membranes^16^,^17^,^18^,^19^,^20^ or with microfluidic structures^21^,^22^, or inertial focusing^23^. However, these techniques require additional sample preparation upstream and/or also have low specificity (0.1% to 1% purity).

Regardless of the CTC collection approach, the current standard for enumerating CTCs involves immunofluorescence staining to visualize specific markers for CTCs and WBCs – a method with significant limitations. While this standard is evolving, in general, cytokeratin (CK) and/or EpCAM markers are used to identify CTCs while CD45 is used for WBCs^11^. White blood cells staining positive for both CD45 and CK may be isolated^24^, and have been identified as granulocytes with additional CD66b staining^25^,^26^ (Supp. Fig. 4). On the other hand, some large cells negative for both CD45 and CK have also been reported. Such double staining or lack of any CK/EpCAM stain makes classification of these cells difficult^10^,^27^,^28^. In addition, several experimental factors can affect immunofluorescence analysis. These include variation in the antibodies used (sensitivity, clones, fluorophores), staining protocol, and lastly the image analysis methodology. In addition to these experimental issues, immunostaining based enumeration is time-consuming and costly. Simple analysis methods would enable a rapid and low cost count for more efficient use of downstream resources (e.g. for genetic analyses) on samples with large cell burdens.

Stains that aid in identifying malignant cells independent of immunostaining already exist. Cytopathologists are trained to interpret the cytomorphological features of cells using these stains. For example, the Papanicolaou (Pap) stain is a routinely used staining method for cancer diagnosis in human cytology^29^. The advantage of the Pap stain relies on its ability to yield detailed information regarding nucleoli and chromatin pattern in the tumor cell. This information is often sufficient to determine whether cells are benign or malignant. Clinical decision-making is often based on these features of a biopsy or cells present in body fluids as determined at the initial cytological assessment. Re-evaluation of the tumors during disease progression is not typically performed, because of the invasive nature of excisional biopsy, and also because the tumor may not be readily accessible. Cytological analysis of CTCs that are continuously shed by tumors into the bloodstream could provide real-time and more representative information on tumor evolution, treatment effectiveness, and cancer metastasis risk. Unfortunately, many CTC technologies cannot provide intact, free-floating CTCs suitable for cytological staining. This prevents compatibility with existing automated laboratory equipment and requires cyto-technicians to be trained on new protocols^30^. Even for separation approaches that isolate cells freely in solution, concentrating the cells into a smaller liquid volume suitable for cytology requires extra processing steps to collect the cells onto glass slides, leading to potential cell loss^31^. Furthermore, the low purity of many cell isolation techniques can confound downstream analysis. Therefore, a high purity platform that can prepare CTCs in a small volume would be ideally suited to enable such cytological studies directly on blood biopsies.

Such a platform could also be beneficial for fluorescence *in situ* hybridization (FISH) assays to make use of CTCs to aid in treatment selection. For example, oncogenic fusion genes consisting of *EML4* and *ALK* have been identified in a small subgroup consisting of ~3–7% of all NSCLC tumors^32^. Recent studies have demonstrated that detecting *ALK* rearrangements can be of clinical value for physicians to select more effective therapies. Indeed *ALK* rearrangements have been shown to be associated with positive response to targeted therapies such as the FDA approved drug crizotinib, a small-molecule inhibitor of the *ALK* tyrosine kinase^33^. Conversely *ALK*-positive patients have shown no benefit from the use of erlotinib. Detecting such rearrangements directly from the blood and in real-time would provide the clinician with a more accurate picture of the cancer, to better personalize the treatment. Other CTC technologies have demonstrated compatibility with FISH assays, for the detection of the *ALK* rearrangement in lung CTCs^17^,^18^ or to analyze the androgen receptor (AR) and *MYC* genomic copy number in patients with castration resistant prostate cancer^34^. However, these technologies generally require a custom filter-adapted FISH (FA-FISH) protocol^18^ or modified imaging equipment, which limits adoption by cytogenetics laboratories.

Our group previously introduced the Vortex Chip, which uses microfluidic vortices to achieve size-based and surface-label-free separation of CTCs from whole blood at high purities (>75% purity)^35^. Here, we combine the Vortex CTC enrichment system with an inline optical measurement and automated image analysis system to perform the label-free counting and morphological classification of all isolated cells (Fig. 1). We leverage the ability of this method to concentrate and easily release cells in a small volume (300 μL) to achieve facile integration with standard cytological and cytogenetic analysis workflows, both of which further improve the accuracy of analysis.

## Materials and Methods

All experiments were performed in accordance with relevant guidelines and regulations.

### Microfluidic Vortex HT device design and fabrication

**Figure 1:**
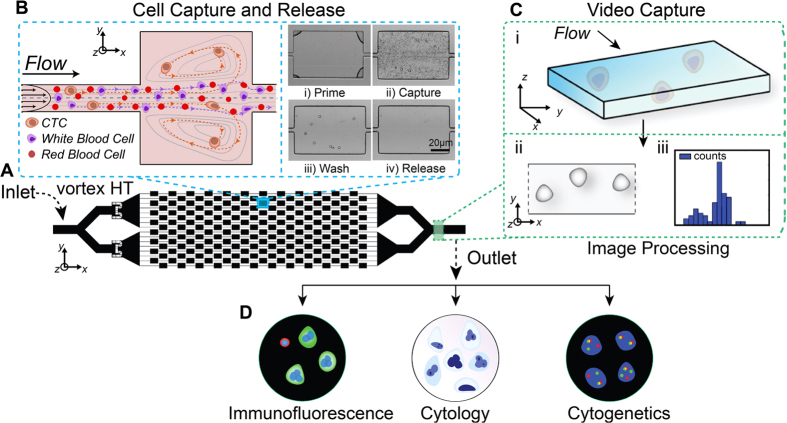
Label-free isolation and enumeration of CTCs followed by downstream analysis. (**A**) The Vortex HT chip is used to capture CTCs in vortices formed in microfluidic reservoirs. (**B**) After a priming step, CTCs are selectively trapped by size in the microscale vortices, RBCs and WBCs are washed away with a clean buffer, then remaining larger trapped cells are released into well plates or slides. (**C**) A region of the outlet channel is recorded to image the released cells in-flow for enumeration. An algorithm is used to process the video, determine morphological features of cells collected, and enumerate based on morphological thresholds. (**D**) The output from the video analysis allows the user to determine whether to perform downstream analysis. This could include immunofluorescence, cytology and cytogenetic analyses.

Microfluidic devices were fabricated using common polydimethylsiloxane (PDMS) replica molding processes^35^. Briefly, standard lithographic techniques were used to produce a mold from a silicon master wafer spin-coated with KMPR 1050 (MicroChem). PDMS chips were produced from this mold using Sylgard 184 Elastomer Kit (Dow Corning Corporation) with a cross-linker to polymer ratio of 1:10. To fabricate the channels, PDMS and glass were both activated by O_2_ plasma (Technics Micro-RIE series 800, 500 mTorr, 80 Watts, 30 sec) before being bonded together. The Vortex HT Chip used for cell trapping is composed of 192 reservoirs, with 16 channels in parallel and 12 reservoirs per path (Fig. 1A). Each path includes a single straight high-aspect ratio channel (*W*_*C*_ = 40 μm, *H* = 70 μm) composed of 12 reservoirs (*W*_*R*_ = 480 μm, *L*_*R*_ = 720 μm) located every 1 mm downstream. Filters located at the inlet prevent channel clogging by cell aggregates or debris present in blood samples.

### Microfluidic Vortex HT device operation

Two syringes on two separate syringe pumps (Harvard Apparatus PHD 2000) were connected to the device inlets to achieve rapid solution exchange^35^,^36^. One syringe contained the 10X diluted blood sample, while the second syringe was filled with Phosphate Buffered Saline (PBS, pH 7.2, Gibco) as a washing buffer (Fig. 1B). Before sample infusion, the channels are primed and the vortices are stabilized by flowing PBS at 8 mL/min. The cell sample was then injected in the device at 7 mL/min while the PBS flow is lowered to 1 mL/min. After sample injection, PBS was flowed into the device at 8 mL/min to wash out unstable blood cells. Finally, by lowering the buffer flow rate, vortices were dissipated and the cells trapped in the vortices were released from the reservoirs and collected in a volume of 300 μL for downstream analysis.

### Cell culture and cell spiking

All cell lines used for characterization were grown to 30–40% confluence at 37 °C in a humid atmosphere of 5% CO_2_. MCF7 (breast carcinoma, ATCC) cells were cultured in EMEM medium supplemented with 10% FBS and 1% penicillin/streptomycin (Invitrogen) and containing 0.01 mg/ml human recombinant insulin. A549 (Squamous non-small cell lung carcinoma) cells were cultured in RPMI medium supplemented with 10% FBS and 1% penicillin/streptomycin (Invitrogen). Adherent cells were dissociated with 0.25% (w/v) Trypsin-EDTA (Gibco), resuspended in full medium and spiked (200–500 cells) in 5 mL Dulbecco’s PBS (DPBS, without calcium, magnesium) or in 5 mL healthy blood which was diluted 10x in DPBS prior to injection through the device. The cell sample was processed as indicated above in the Microfluidic Vortex HT Device Operation section and the captured cells were released into a 96-well plate as described below. To increase capture efficiency, sample flow-through was collected in a separate tube and reprocessed through the device for one or more additional cycles.

### Patient recruitment, blood collection and processing

Patients with metastatic cancer were recruited according to protocols with informed consent approved by Institutional Review Boards (UCLA-IRB#11-001798 and Stanford-IRB Protocol#5630) from the Department of Radiation Oncology and the Department of Hematology and Oncology at the Geffen School of Medicine at UCLA, and from the Stanford University School of Medicine, respectively. Blood samples were drawn from 54 patients, diagnosed with metastatic non-small cell lung cancer (NSCLC, N = 32) and metastatic breast cancer (N = 22), as well as 12 healthy age-matched volunteers (Supp. Table 1). Peripheral blood was collected into 10 mL EDTA-coated tubes (BD Vacutainer), transported and processed at room temperature within 4 hours of the blood collection, as described in the Device Operation section above.

### High-speed, label-free cell imaging and automated cell analysis script

A high-speed camera was used to collect videos of cells during their release from the vortices and as they flowed past the imaging region (a portion of the outlet channel). The resulting video images were then analyzed using a custom image processing algorithm (Supp. Fig. 1). Videos were collected using a 10X brightfield Nikon objective lens mounted on a Phantom v2010 high-speed camera and controlled through a Phantom Camera Control and Software (Vision Research Inc). For each incident cell, a collection of images (256 × 376 pixels) was captured (6006 frames per second) for a total of 15 seconds. The cells appeared 6 times in the viewing window to ensure that none were missed. The focal plane was manually adjusted prior to sample injection, using the walls of the exit channel area as a reference. To initiate the release of the trapped cells, the flow rate was stopped and 15 s of video was recorded concomitantly. Cells remained in focus when recording a region downstream near the outlet because here we avoid a large hydrodynamic pressure buildup during operation. The depth of focus of the 10x Nikon objective used was 8.5 μm and, given the large depth of focus, small shifts out of the focal plane were not found to significantly affect the size calculations when imaging of these cells.

**Figure 2:**
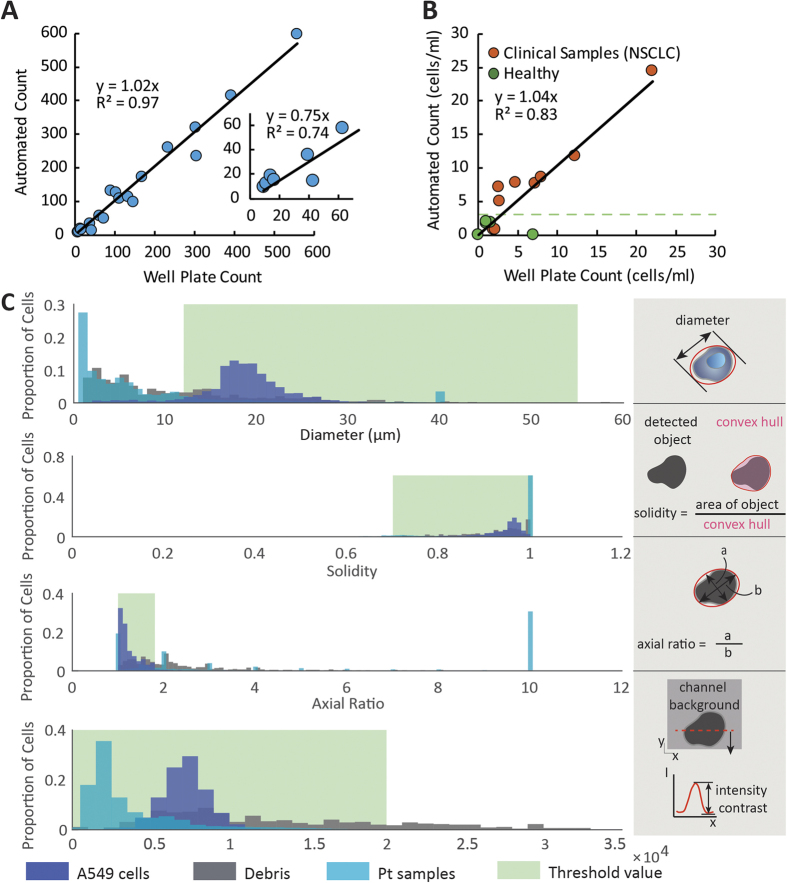
In-flow label-free counting of large cells. (**A**) The in-flow counting algorithm was validated with A549 cell lines. 10–600 cells were initially spiked into 5 mL of PBS and processed. A correlation of R^2^ = 0.97 was found between the number of cells identified per the algorithm and the number of cells manually counted in the well plate. Inset axes show cells per mL. (**B**) This method was used to enumerate 9 NSCLC patient samples (L1–9), and 5 samples from healthy donors (H1–5). Only cells larger than 12 μm were counted for the well plate. The counting criteria for the cells imaged in-flow included ranges for cell diameter, circularity, axial ratio, and specific pixel intensity distribution defined with A549 cells. As indicated with a green dotted line, a healthy threshold can be calculated from healthy donors at 3.15 cells/mL (mean + 2SD) (**C**) The counting criteria for the cells imaged in-flow included threshold ranges for cell diameter, solidity, axial ratio, and specific pixel intensity distribution. The thresholding order is given in the four histograms. All parameters for all objects that were detected is shown before thresholding was performed. Clinical blood samples had a larger proportion of debris compared to A549 spiked in blood.

The image processing algorithm identifies cells by their morphology. Objects are detected using a background subtraction method. Briefly, the intensity values of an empty frame are subtracted from each ensuing image of the video, which eliminates the background image and yields traces of incident particles in the frames in which they appear (Supp. Fig. 1). In our algorithm that detects object boundaries first, each image is scaled by a factor of 3 with interpolation. The images are converted to binary, and the particle traces are then filled with morphological closing (dilation followed by erosion) to be measured for their diameter, solidity (area/convex hull, where convex hull is the smallest enclosed convex region around the object), axial ratio (major axis/minor axis), and cell interior pixel intensity distribution. Data were thresholded for diameter greater than 12 μm and less than 55 μm. A detailed description of these parameters is provided in Fig. 2C. These parameters were determined during control experiments with lung cancer cell lines. The detected objects are displayed in a matrix. These results give us information concerning the number of cells collected and their morphologies.

### Immunofluorescence cell staining and enumeration

After collection in a 96-well-plate (CELLSTAR, Greiner Bio-one), cells were fixed with 4% paraformaldehyde (Boston BioProducts) and permeabilized with 0.4% Triton X-100 (Sigma-Aldrich) and stained with 4′,6-diamidino-2-phenylindole (DAPI, Invitrogen), CD45-PE (Clone HI-30, Becton Dickinson (BD), Pan-CK-FITC (Clone CK3-6H5, Miltenyi Biotec), CK CAM5.2-FITC (Clone CAM 5.2, BD), and Pan-CK-FITC (Clone AE1-AE3, eBioscience) to target CK 1–8, 10, 14–16, 18–19. After staining, each well of collected cells was imaged using a Photometrics CoolSNAP HQ2 CCD camera mounted on an Axio Observer Z1 microscope (Zeiss), with an ASI motorized stage operated with Zen software. Cells were manually classified by two independent trained readers and enumerated using a criterion (Supp. Fig. 4)^25^ that is based on three categories: debris, WBCs, or CTCs. In general, debris was characterized by irregular, jagged shapes or dark outlines under bright-field microscopy. Among the WBCs, there were (i) CK−/CD45+/DAPI+ cells, (ii) doubly-stained CK+/CD45+/DAPI+ cells generally corresponding to CD66b+ activated granulocytes^25^, and finally (iii) DAPI+ only cells distinguished by lobular or segmented granulocytic nuclei, small nuclei (<9 μm), and/or small N:C ratios. CTCs were primarily characterized as (i) CK+/CD45−/DAPI + cells or (ii) DAPI+ only cells with a large nucleus (>9 μm) and large N:C ratio.

### Cell collection for cytological analysis

A first benchtop collection system for preparing cytology slides was developed for testing, based on a removable PDMS well. PDMS was poured into a 6-well plate at a cross-linker to polymer ratio of 1:10. After curing, the PDMS was removed and punched with a 17-mm biopsy punch (Supp. Fig. 5). The resulting ring was bonded to a microscope slide (Fisherfinest Premium Plain) using the same plasma bonding techniques as for the microfluidic devices. The CTCs were collected directly onto the ring containing 95% ethanol. The slide was then agitated on a shaker plate overnight at room temperature. After evaporation, 1 mL of Clearprep cytology solution was added to the slide, and let to dry. The PDMS insert was then removed and the slide processed for cytological staining.

A second collection method, using commercially available materials, was evaluated and consisted of a removable media chamber attached to a standard glass microscope slide (Lab-Tek II chamber slide system, Nunc). CTCs were collected in PBS directly in one of the chambers, the slide was centrifuged at 800 g for 10 minutes and the cells were let to attach for 1 hour at room temperature. The cells were then fixed for 20 minutes with Clearprep fixative (Resolution Biomedical), the fixative was removed carefully and replaced with an equal volume of a Clearprep cytology solution, and the slide was left to dry overnight before staining. Variations around this protocol were tested, including the use of different fixatives, of coated chamber slides (fibronectin, Poly-D-Lysine) and different incubation times.

Slides were stained on a Sakura Tissue-Tek DRS 2000 (Sakura) following the UCLA Cytopathology Papanicolaou staining protocol used for clinical samples. Briefly the protocol consisted of incubations in Hematoxylin, Orange G, and Eosin Azure, with corresponding washes (Thermo Scientific) (Supp. Text 1). Slides were then mounted with mounting medium (Richard Allan Scientific), coverslipped and examined by a cytopathologist blinded to the immunofluorescence CTC counts. For cell line characterization experiments, all conditions were tested in duplicate. Following PAP staining and mounting, the slides were imaged with a microscope, the cells were counted and a recovery rate (in %) was calculated as the number of cells counted after PAP stain divided by the number of cells seeded at t = 0 on the slide.

Preparation of cytology slide with Cytospin was evaluated in parallel using a protocol from the UCLA Cytopathology Department. Briefly, single cytofunnels were assembled with white filter cards (Shandon), loaded with 200 μl of a cell suspension containing 300 MCF7 cells, and ran in the cytocentrifuge (Shandon, Cytospin III) for 10 minutes of 500 rpm. After disassembling the fixtures, cell spots were fixed for 10 minutes with 95% ethanol, the slides left to dry overnight at RT, then stained and cells counted.

### Cell collection for cytogenetic analysis

Positively charged slides (Fisherbrand Superfrost Plus) were used for FISH analysis in combination with the first collection system described previously for cytological analysis (Supp. Fig. 5). Cells were released into the PDMS ring with 95% ethanol and then placed in a fume hood to allow for complete evaporation. The PDMS ring was removed once the slide was dry. Slides were processed using the standard FISH protocol provided by the vendor Abbott Molecular (Des Plaines, Illinois) using their FDA-approved Vysis LSI ALK Break Apart Rearrangement FISH Probe Kit. After hybridization, the slides were analyzed under a fluorescence microscope (Zeiss Axiophot) equipped with multicolor filters and 63X and 100X oil immersion objective lenses. All images were taken at 100X with 1.6X magnification.

The analyzed cells were classified as: (a) negative for *ALK* rearrangement with two copies of chromosome 2p, (b) negative for *ALK* rearrangement with 3 or more copies of chromosome 2p (polysomy), (c) positive for *ALK* rearrangement and positive for *ALK* rearrangement along with three of more copies of 2p (polysomy), and (d) ambiguous signals pattern and uninterpretable. As defined by the ALK FISH probe Signal Enumeration Guide: (1) *ALK*-negative nuclei show two yellow (Red-Green) signals, (2) *ALK*-negative and polysomy of 2p nuclei show three or more yellow (Red-Green) signals, (3) *ALK*-positive nuclei show many variations of the signal pattern but all show one or more copies of split red-green signals consistent with *ALK*-rearrangement. (4) Uninterpretable nuclei had unclear or unresolvable probe signals probably caused by pyknotic nuclei or the presence of background noise.

## Results

### In-flow and label-free counting of large circulating cells

Because of the high purity isolation achieved with the Vortex HT Chip, we investigated counting of all isolated large cells as a measure of abnormal malignant processes^25^,^35^. A high large cell count, whatever the origin of these cells, could then indicate that downstream label-based enumeration assays would be warranted. For this purpose, we developed an in-flow counting system to enumerate the number of cells trapped and released by the Vortex HT chip before their collection at the chip outlet (Fig. 1). The system achieved high-speed brightfield imaging of released cells followed by image analysis to identify and extract morphological features for each cell. We trained the image analysis algorithm to identify cancer cells by first capturing A549 lung cancer cells spiked in PBS in the vortex traps and enumerating the released cells, before progressing to clinical patient samples. Accepted ranges for cell diameter, solidity, axial ratio and pixel intensity distribution were determined based on the in-flow morphology of A549 cells. Cell diameter ranged from 12 to 55 μm, solidity ranged from 0.7 to 1, axial ratio ranged from 1 to 1.8, and the pixel intensity range within the cell was 0–20,000. Following this training and to evaluate the performance of our label-free approach, A549 cells (10 to 600) were spiked into 5 mL of PBS and processed through the Vortex HT Chip. The captured cells were simultaneously enumerated in-flow and after collection in a well-plate followed by DAPI staining. The DAPI staining was used to determine location of the cell, and the brightfield image was used to measure the size of the cell. Figure 2A indicates a high correlation (R^2^ = 0.97) and unity relationship (y = 1.02x) between the number of cells counted with in-flow counting using an automated script and the number of cells enumerated after staining in the well-plate, and supported the accuracy of our counting method.

We next applied this approach to counting the cells in NSCLC patients and age-matched healthy blood samples. Samples from nine NSCLC patients and five healthy controls show a strong linear correlation between automated cell counts and well plate counts (Fig. 2B), with a slight overcounting bias for the automated count (y = 1.04x). A gallery of cells imaged in-flow is shown in Supp. Fig. 3. Cell counts from healthy samples can be used as a threshold to define patients with abnormal numbers of large cells above this healthy background. Using automated counting, a healthy threshold can be defined at 3.15 cells/mL (mean + 2SD), leading to 7/9 (77.8%) of NSCLC patients with automated cell counts above the healthy threshold. Using well-plate cell counts, the healthy threshold can be defined at 7.7 cells/mL, with 3/9 (33%) of the patients above the threshold. For both counting approaches, patients L5, L8, and L9 were above threshold while patients L3 and L6 were below. Patient L7 is just below the threshold using well-plate-counts (7.25 cells/mL) but above the threshold according to automated-counting (7.63 cells/mL). These data validate the ability of automated-counting to yield rapid label-free enumeration of large cells following Vortex trapping, which are present at higher levels in NSCLC patients.

### Cytological analysis of circulating tumor cells

**Figure 3:**
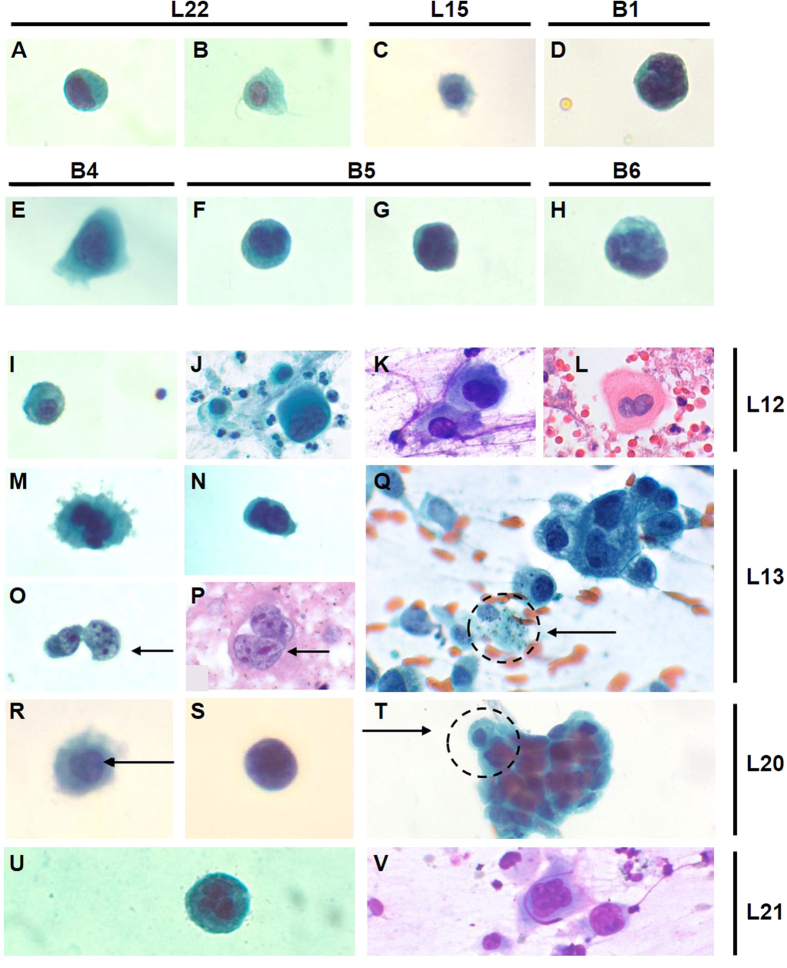
Cytological analysis of CTCs from NSCLC (L) and breast (B) cancer patient blood. Cell Morphology. (**A**–**H**) Gallery of cells isolated with the Vortex Chip, stained with a conventional Papanicolaou procedure and classified as highly atypical by a cytopathologist due to nuclear morphologies (e.g. hyperchromasia and chromatin margination, irregular nuclear membrane, and sometimes multinucleation) and high nuclear to cytoplasm ratios. (**I**–**V**) Cells from patients L12, L13, L20, L21 were compared to their primary tumor biopsies or other body fluids to identify morphological similarities. (**I**–**L**) Patient L12: The cell detected from patient L12 (**I**) revealed binucleation, which was recapitulated in cells found from a Pap stain (**J**), MGG stain (**K**) of the pleural fluid, and H&E (**L**) of the primary tumor. (**M**–**Q**) Patient L13: Cells isolated from patient L13 (**M**–**O**) had a very high N/C ratio and a distinct chromatin margination pattern that was recapitulated in cells from a Pap (**P**) and H&E stain (**Q**) of a fine needle aspirate. (**R**–**T**) Patient L20: Both the circulating cells from the liquid biopsy (**R**,**S**) and the cells from the core needle biopsy (**T)** had irregular nuclear contours and hyperchromasia. (**U**,**V**) Patient L21: Very large cells with multinucleation were seen in sample L21, both in circulation (**U**) and in Diff-Quick stain of tumor biopsy (**V**).

**Figure 4:**
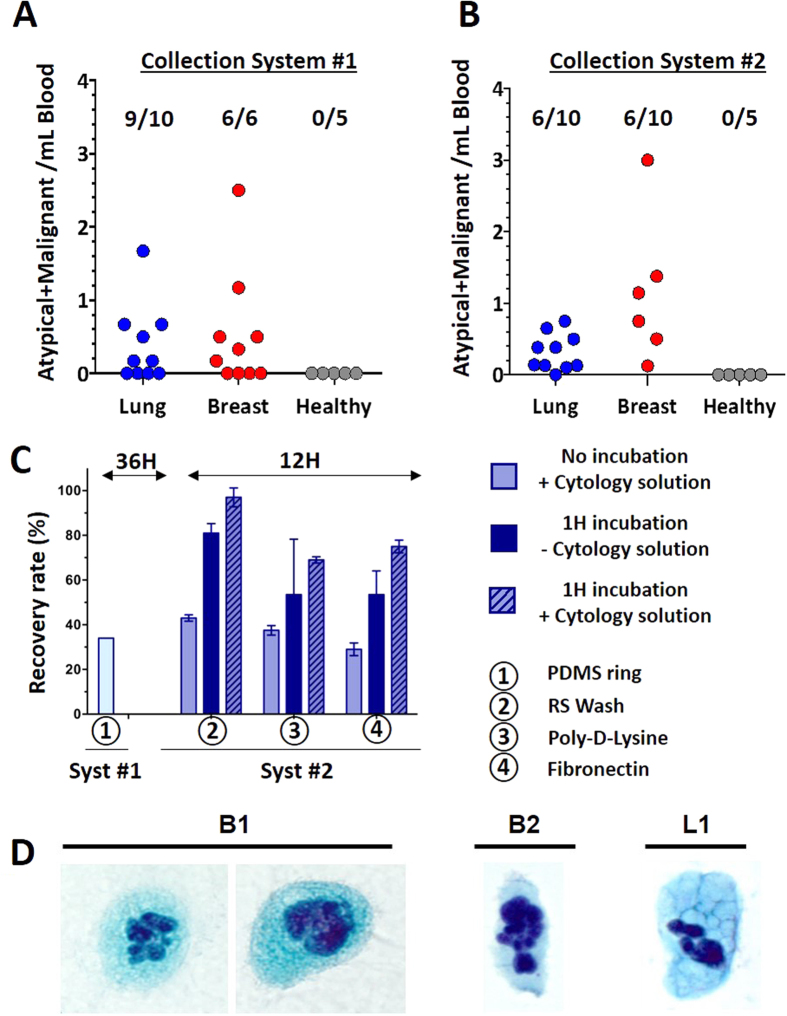
Cytological analysis of CTCs from NSCLC (L) and breast (B) cancer patient blood. Cell Enumeration. (**A**) Collection System #1: Over 16 samples were Pap stained, and malignant cells were detected in 9/10 NSCLC patients and 6/6 breast cancer patients, while none were detected in 5 healthy donors. Processing failed (peeling of the cell layer) for 7 samples. (**B**) To solve the cell layer peeling, a new Collection System #2 was tested with 20 new samples: Over 20 samples, malignant cells were detected among 6/10 NSCLC patients and 6/10 breast cancer patients, while none were detected in 5 healthy donors. (**C**) Evaluation of the effect of different parameters (fixatives, surface of chamber slides, incubation time, use of cytology solution) on cell recovery after Pap staining using an MCF7 breast cancer cell line as a model. An optimal recovery rate and staining quality was achieved with RS treated glass slides, for a recovery rate of 97% and a processing time of about 12 hours, compared to 34% recovery and 36 hours processing using the initial protocol. (**D**) Using those optimal conditions, CTCs were isolated from patient whole-blood samples, Pap stained, and classified as malignant by a cytopathologist due to: abundant granular cytoplasm with large pleomorphic, lobulated nuclei (B1), foamy cytoplasm and large pleomorphic hyperchromatic nuclei (B2), and enlarged nuclei with hyperchromasia and coarse chromatin (L1).

We tested two methods to integrate Vortex-based CTC isolation with the downstream workflow for cytological studies. In the first CTC collection system, CTCs released from the Vortex chip were collected in a PDMS well reversibly bonded to a microscope glass slide (Supp. Fig. 5). Using this device, CTCs isolated from ten NSCLC and six breast cancer patient samples were collected and stained with conventional Papanicolaou stain (Pap stain) (Fig. 3, Supp. Text 1). Malignant cells were detected in 9 of the 10 lung and 6 of the 6 breast cancer cases, while no malignant cells were detected in 5 healthy donors (Fig. 4A). Comparison of Pap-stained CTCs isolated from four NSCLC patients to their corresponding primary tumor biopsies stained with hematoxylin and eosin (H&E) or May-Grunwald Giemsa (MGG), revealed conserved morphological features (Fig. 3I–V): The circulating cell detected from patient L12 (I) reveals a large size and binucleation, which is recapitulated in cells found from a conventional Pap stain (J) and MGG stain (K) of pleural fluid from this patient, as well as the H&E stain (L) from the cell block. Cells isolated from the blood of patient L13 (M, N, O) have a very high N/C ratio and a distinct chromatin margination pattern that is recapitulated in cells from a Pap (P) and H&E stain (Q) of the fine needle aspirate. For patient L20, both the circulating cells from the liquid biopsy (R, S) and the cells from the core needle biopsy (T) have irregular nuclear contours and hyperchromasia. Finally, very large cells with multinucleation are seen in patient L21, both in circulation (U) and in a Diff-Quik stain of tumor biopsy (V).

Although atypical cells were detected in 15/16 patient samples successfully processed and stained, some samples (1 NSCLC, 5 breast) peeled off during the PDMS ring removal due to lack of adhesion of the cell embedded layer on the slide and were not usable for quantitative counting following Pap staining (Supp. Table 1).

To limit this cell loss during processing and staining and to improve the adherence of the cell layer, as well as to accelerate the overall processing, we evaluated the effects of different parameters on cell recovery after Pap staining using a breast cancer cell line (MCF7) as a model. We replaced the PDMS ring collection system by a commercially available chamber glass slide. Parameters tested included fixatives (95% ethanol, Clearprep fixative), growth surface of chamber slides (RS wash, fibronectin, Poly-D-Lysine), incubation time (1-hour vs no incubation), and cytology solution (Fig. 4C). Improved cell recovery (ranging from a 1.23 to a 1.97-fold increase) was achieved when the cells were incubated for 1 hour on the glass slide. The addition of cytology solution further increased the recovery rates and this effect was more pronounced when the Clearprep fixative was used as opposed to 95% ethanol. The optimal recovery rate and staining quality were achieved with RS treated glass slides, for a recovery rate of 97%, compared to 69% and 75% for Poly-D-Lysine and fibronectin coated slides respectively, and 34% cell recovery using the initial protocol. Processing time for this new collection procedure was about 12 hours compared to more than 36 hours for the original protocol.

As a final validation, the chamber slide protocol was tested in parallel with Cytospin. Cytospin is a conventional cytology technique that uses a high speed centrifuge to concentrate cells from biopsies on a glass slide in a uniform monolayer. The monolayer distribution enhances the morphological appearance of the cells present and facilitates cytological staining and identification of atypical cells. In this comparison, 300 MCF7 cells were either seeded on a chamber glass slide, or loaded into the cytology sample funnel (Supp. Fig. 5). A significant cell loss was observed with the Cytospin concentration method, with only 25% cell recovery onto the glass slide, as opposed to 71% for the chamber glass-slide method. This low cell recovery with Cytospin can be explained by the low number of cells (300) loaded on the cytofunnel. This number of cells was chosen to reflect the low cellularity of the samples after processing through our CTC platform. The recommended number of cells for Cytospin is typically in the 1–2 × 10^5^ range. This side-by-side comparison highlights the challenge of using Cytospin for rare CTC concentration and the need for a specific CTC concentration method for cytology purposes, which is one of the important points of our manuscript which we believe will assist the community.

Using those optimal conditions (RS wash glass slide, incubation of the cells for 1 hour, Clearprep fixative, cytology solutions), we then collected and Pap-stained an additional set of CTCs isolated from patients with NSCLC (N = 10) and breast cancer (N = 10), all in Stage IV (Fig. 4B). After Pap staining, malignant and atypical cells were identified, enumerated by a cytopathologist and cell counts normalized per mL of blood processed. Using Pap staining, malignant cells were identified in 6/10 (60%) of lung and in 6/10 (60%) of breast cancer patients while none were detected in 5 healthy volunteers. No cell layer was peeled off during the staining procedure as observed in the first approach. Samples were considered positive when cell count was greater than the threshold as determined from the healthy volunteer cohort (as mean + 2SD). Using this threshold (0 atypical + malignant cells/mL), 60% (6/10) of lung and 60% (6/10) of breast cancer samples were considered positive for CTCs (Fig. 4B). As a comparison, another blood tube from each patient was processed the same way, but with the cells being collected in a well-plate for conventional marker-based enumeration (CK, CD45, DAPI) as previously described by Che *et al.*^25^. Detailed explanations of these cell classifications with accompanying image galleries are shown in Supp. Fig. 3. Using such immunostaining and classification criteria, more CTCs were found in lung (mean: 2.58 CTCs/mL, range: 1.00–5.50 CTCs/mL) and breast (mean: 3.95 CTCs/mL, range: 1.17–11.17 CTCs/mL) cancer samples than in healthy controls (mean: 0.72 CTCs/mL, range: 0.25–1.17 CTCs/mL) (Supp. Fig. 3C). Using the healthy threshold of 1.46 CTCs/mL (mean + 2SD), approximately 80% (8/10) of lung and 80% (8/10) of breast cancer samples, were considered positive for CTCs by immunofluorescence staining.

### Cytogenetic analysis of circulating tumor cells

**Figure 5:**
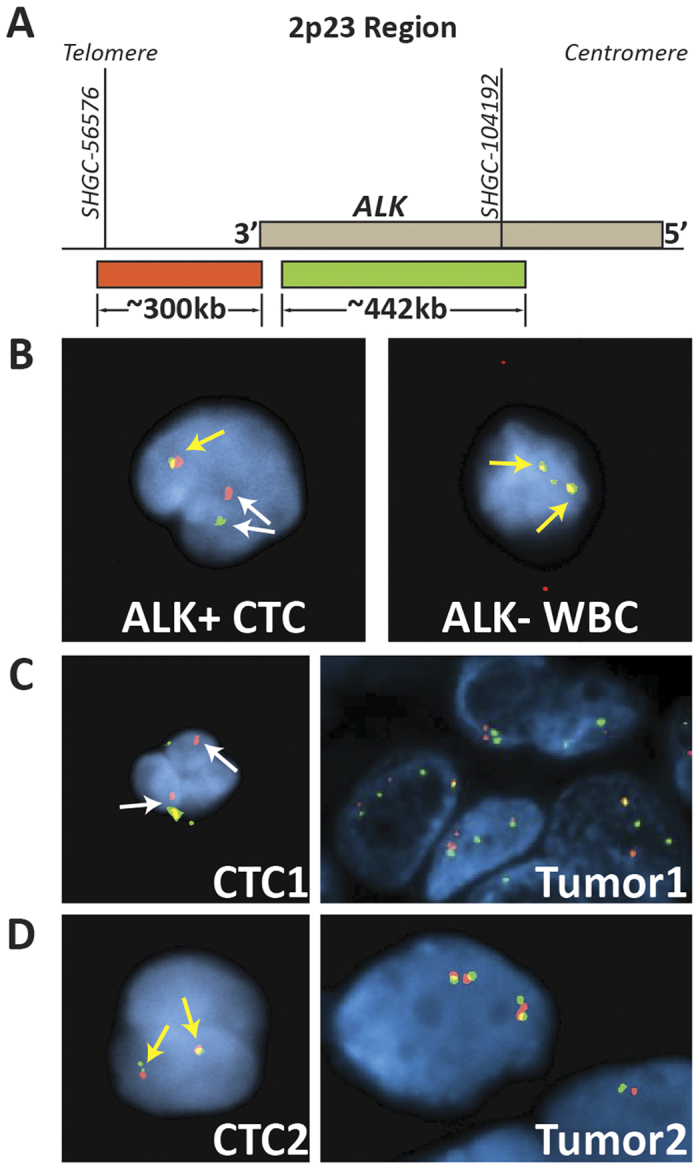
Cytogenetic analysis of Vortex isolated circulating tumor cells. (**A**) Schematic of Abbot Molecular LSI ALK Break Apart Rearrangement Probe Kit (www.abbottmolecular.com). (**B**) Images of (left) ALK+ CTC isolated from a NSCLC patient sample and (right) ALK- WBC from a healthy donor. Non-rearranged ALK gene is identified by red and green probes next to each other or overlapping for a yellow signal (yellow arrows). ALK rearrangements are identified by split green and red signals (white arrows). (**C**) ALK+ patient. (Left) Isolated CTCs have ALK probe patterns that are similar to those from the primary tumor (right): CTC1 and Tumor1 both show ALK rearrangement as well as polysomy, which is indicated by the multiple probe signals of each color in each nuclei. (**D**) ALK- patient. CTC2 and Tumor2 both exhibit normal ALK signals.

Fluorescence *in situ* hybridization (FISH) is a gold standard diagnostic test used in the clinic for the detection of genetic aberrations including rearrangements (translocations, inversions) and changes in gene copy number associated with cancer^32^,^33^. As a proof of principle, we assessed the feasibility of characterizing the anaplastic lymphoma receptor tyrosine kinase (ALK) gene rearrangement by FISH in CTCs isolated from patients with non-small cell lung carcinoma (NSCLC) (Fig. 5A–B).

Blood samples were collected from one ALK-positive patient and ALK-negative patient, processed through the Vortex chip and CTCs were collected on a glass slide using the standard FISH procedures. The fluorescence signal patterns obtained from the cells collected from the liquid biopsy were compared to FISH results performed previously on the primary tumor biopsy. Notably, the isolated CTCs displayed similar *ALK* probe patterns when compared to the corresponding primary tumors (Fig. 5C). CTCs and tumor sections both showed *ALK* rearrangement as well as complex signal patterns, as evidenced by the multiple probe rearrangements and polysomy in the nuclei. However, no-rearrangements-positive signal patterns were observed for the cells collected from the ALK negative patient and its primary tumor counterpart (Fig. 5D).

## Discussion

We have demonstrated the utility of Vortex trapping technology as a platform to enable extraction of CTCs and interfacing with downstream assays of clinical importance. Our cell capture approach enables the coupling of label-free cell enumeration in-flow based on bright field images with various standard assays downstream, such as cytology and cytogenetics. In-flow microfluidic cytological staining could also enhance such assays in the future, enabling rapid counts^37^. A simple count of tumor-associated large circulating cells obtainable within minutes after processing could avoid costly immunofluorescence-based enumeration as used currently, as well as indicate downstream assays for the subset of samples with sufficient numbers of cells, also avoiding wasted time and costs.

All cells collected can be counted in-flow, without the need for any cell-specific labels and remain unaltered, allowing cells to be collected off-chip for further characterization assays, such as cytology, cytogenetics, genomic profiling, transcriptomic and drug assays, and even conventional immunofluorescence. More importantly, these results introduce label-free analysis as a potential method to rapidly identify patient samples with high cell counts directly compatible with downstream assays (e.g. cytology, FISH, genetic analysis, or further immunostaining), or conversely, limit the use of valuable resources on samples without significant diagnostic cells. Although these initial data are promising, additional patient samples from a variety of cancer types and cancer stages are needed to validate the approach further. Further, direct single-cell comparisons between morphological and immunofluorescence staining for CTCs would be interesting to investigate in the future, and should be enabled by high-speed imaging cytometry technology^38^.

The large cytological dataset of over 35 patients revealed that circulating tumor cells share striking cytomorphological features with malignant cells from other body fluids. The most common types of features include abundant granular cytoplasm, foamy cytoplasm, large pleomorphic and lobulated nuclei, large pleomorphic hyperchromatic nuclei, as well as nuclei with hyperchromasia and coarse chromatin. Similar observations have been made with filter-based approaches or high-throughput imaging on a glass slide^30^,^31^, but without cell enumeration and with the need for red blood cell lysis and a staining protocol which differ from the standard cytopathology workflow. Further clinical studies are needed in collaboration with cytopathologists at multiple sites to make any conclusion on the clinical use of liquid biopsies followed by standard cytological analysis for cancer diagnosis and prognosis^30^.

Further clinical testing using our label-free approach from numerous patient samples of different tumor types and different cancer stages would allow the development of an improved database of both CTC brightfield and Pap images^30^. This database could then be analyzed with machine learning algorithms to define new biomarkers for cancer diagnosis and prognosis, such as the number of cells above a certain size or number of cells with a distinct morphology, leading to improved differentiation from large cells present in healthy patients. A simple label-free count of abnormal cancer-associated cells could be more widely adopted for cancer prognosis, and also serve to direct downstream assays when most likely to provide clinically-actionable information – making headway towards personalized medicine that can be largely deployed.

Similarly, we have demonstrated the compatibility of Vortex CTC enrichment with FISH assays. More clinical assays are warranted to validate the use of FISH for CTCs and liquid biopsies to define the corresponding threshold for identification of a patient that would respond to targeted therapies based on chromosomal aberrations. Detecting ALK translocations in cells directly isolated from the blood of an ALK-positive patient not only confirms the malignant nature of the isolated cells, but also supports the compatibility of CTCs isolated with our label-free approach with commercially available, FDA-approved FISH assays. FISH analysis of CTCs can be a valuable, noninvasive surrogate for routine tumor cytogenetic profiling and may provide additional information to help predict a patient’s outcome, identify patients eligible for targeted drug therapy and/or monitor response to treatment. Detection of CTCs harboring a unique ALK rearrangement in ALK-positive NSCLC patient samples was emphasized by Pailler *et al.*^18^, where the CTCs were isolated with the ISET FISH kit (FA_FISH) and enumerated with CellSearch. Data suggest that ALK rearrangements can be reliably detected in CTCs from ALK-positive NSCLC patients and that serial evaluation of the CTCs could be used to monitor response to targeted therapy such as crizotinib and/or to enable detection of resistance markers. In addition, given the specific signal pattern of ALK-rearrangement that was detected only in CTCs and not observed within the tumor samples by Pailler *et al.*, a single tumor biopsy may not be representative of the entire tumor and CTCs that may originate from various metastatic sites may be more informative^17^,^18^. In tumor samples analyzed by FISH, a threshold of 15% ALK- rearranged cells is typically used to define ALK-positive patients. Further clinical studies would now be necessary to define the cutoff value for positive blood biopsies that correlate with response to treatment for example.

Besides cytology and cytogenetics, this label-free collection and enumeration platform is enabling for almost any downstream analysis approach, and allows for rapid and cost-effective decisions concerning the next step of a sample processing. The sample is prepared in a small volume compatible with the standard workflows in the clinical laboratory. This enables for example immunofluorescence staining, targeting Her2, ER, EGFR, PSA, cMET or PDL1 among others, to better profile CTC protein expression and identify new biomarkers, and should be also compatible with single-cell DNA and RNA sequencing. Beyond traditional body fluids analyzed in clinical labs, using a vortex-based approach now opens up the use of blood as a new source of diagnostic cells for the cytopathologist for breast and lung cancer diagnosis.

## Additional Information

**How to cite this article**: Dhar, M. *et al.* Label-free enumeration, collection and downstream cytological and cytogenetic analysis of circulating tumor cells. *Sci. Rep.* **6**, 35474; doi: 10.1038/srep35474 (2016).

## Supplementary Material

Supplementary Information
