# Supplementary material for: Label-free enumeration, collection and downstream cytological and cytogenetic analysis of circulating tumor cells
Source: Sci Rep. 2016 Oct 14;6:35474. doi: 10.1038/srep35474 (PMC5064381; doi:10.1038/srep35474)
Supplement: Supplementary Information [file srep35474-s1.pdf]

Label-free enumeration, collection and downstream cytological and cytogenetic analysis of circulating tumor cells

Manjima Dhar*[1], Edward Pao*[1], Corinne Renier*[3,8], Derek E. Go[1], James Che[1,3], Rosita Montoya[2], Rachel Conrad[2], Melissa Matsumoto[1], Kyra Heirich[8], Melanie Triboulet[8], Jianyu Rao[2,5], Stefanie S. Jeffrey[8], Edward B. Garon[5,6], Jonathan Goldman[5,6], Nagesh P. Rao[2], Rajan Kulkarni[4,5,7], Elodie Sollier[1,3,8], and Dino Di Carlo[1,3,4,5]

1. Department of Bioengineering, University of California, 420 Westwood Plaza, 5121 Engineering V, P.O. Box 951600, Los Angeles, CA 90095, USA.
2. Department of Pathology and Laboratory Medicine, University of California, Los Angeles, Los Angeles, CA 90095, USA.
3. Vortex Biosciences Inc., 1490 O'Brien Drive, Suite E, Menlo Park, CA 94025, USA.
4. California NanoSystems Institute, 570 Westwood Plaza, Building 114, Los Angeles, CA 90095, USA.
5. Jonsson Comprehensive Cancer Center, Los Angeles, CA 90095.
6. UCLA Santa Monica Hematology Oncology, 2020 Santa Monica Blvd, Suite 600, Santa Monica, CA 90404, USA.
7. Division of Dermatology, UCLA Medical Center, 52-121 CHS, Los Angeles, CA 90095.
8. Department of Surgery, Stanford University School of Medicine, MSLS Bldg, 1201 Welch Road, Stanford, CA 94305.

* These authors contributed equally

SUPPLEMENTARY MATERIAL

Supp. Table 1. Summary of all cells collected from patient and healthy donor blood samples. NSCLC stands for Non-Small-Cell Lung Cancer.

1. In-flow counting Study

Patient Info				Well-Plate		Automated
**Patient #**	**Type**	**Stage**	**Sex, Age**	**Vol. (mL)**	**Cells/mL**	**Cells/mL**
L1	Lung, NSCLC, Adeno	IV	F, 91	4	4.75	7.75
L2	Lung, NSCLC, Adeno	IV	M, 52	5	2.6	7.2
L3	Lung, NSCLC Adeno	IV	M, 39	4	2	0.75
L4	Lung, NSCLC, Adeno	IV	M, 82	4	2.75	5
L5	Lung, NSCLC, Adeno	IV	F, 58	6	12.33	11.83

L6	Lung, NSCLC, Squamous	IV	M, 70	6	2.17	0.83
L7	Lung, NSCLC, Adeno	IV	M, 67	8	7.25	7.63
L8	Lung, NSCLC, Adeno	IV	F, 58	2	22	24.5
L9	Lung, NSCLC, Squamous	IV	M, 63	3	8	8.67
H1	Healthy	N/A	F, 52	5	1.6	1.8
H2	Healthy	N/A	M, 24	4	1	1.75
H3	Healthy	N/A	M, 38	4	7	0
H4	Healthy	N/A	M, 25	2	1	2
H5	Healthy	N/A	M, 24	2	0	0

2. Cytogenetics

Patient Info				
**Patient #**	**Type**	**Stage**	**Sex, Age**	**ALK Status**
L10	Lung, NSCLC, Adeno	IV	M, 65	ALK+
L11	Lung, NSCLC, Adeno	IV	M, 63	ALK-

3. Cytology

- Cytology Study, Collection System #1 (Pap only)

Patient Info				Cytology	
**Patient #**	**Type**	**Stage**	**Sex, Age**	**Vol. PAP**	**# Malignant/mL**
L12	Lung, NSCLC, Adeno	IV	M, 64	8	**0.125**
L13	Lung, NSCLC, Adeno	IV	M, 73	8	**0.500**
L14	Lung, NSCLC, Adeno	IV	M, 73	8	**0.125**
L15	Lung, NSCLC, Adeno	IV	F, 79	8	**0.375**
L16	Lung, NSCLC, Adeno	IV	M, 65	7.3	**0.137**
L17	Lung, NSCLC, Squamous	IV	M, 78	7.7	**0.649**
L18	Lung, NSCLC, Squamous	IV	F, 57	9	**0**
L19	Lung, NSCLC, Adeno	IV	M, 49	8	**delamination**

L20	Lung, NSCLC, Squamous	IV	F, 69	8	**0.375**
L21	Lung, NSCLC, Squamous	IV	M, 78	10	**0.100**
L22	Lung, NSCLC, Adeno	IV	F, 80	8	**0.750**
B1	Breast, ER+/PR+/Her2-	IV	F, 59	8	**1.375**
B2	Breast, ER+/PR+/Her2-	IV	F, 55	8	**delamination**
B3	Breast, ER+/PR+/Her2-	IV	F, 59	4	**delamination**
B4	Breast, ER+/PR+/Her2-	IV	F, 71	6	**0.500**
B5	Breast, ER+/PR+/Her2-	IV	F, 75	4	**0.750**
B6	Breast, ER+/PR+/Her2-	IV	F, 51	7	**1.143**
B7	Breast, ER+/PR+/Her2+	IV	F, 37	8	**0.125**
B8	Breast, ER+/PR+/Her2-	IV	F, 69	8	**delamination**
B9	Breast, ER-/PR-/Her2+	IV	F, 46	7	**delamination**
B10	Breast, ER+/PR+/Her2-	IV	F, 44	8	**delamination**
B11	Breast, ER+/PR+/Her2-	IV	F, 66	6	**3**
B12	Breast, ER+/PR+/Her2-	IV	F, 64	8	**delamination**
H6	Healthy	N/A	M, 20	8	**0**
H7	Healthy	N/A	M, 66	8	**0**
H8	Healthy	N/A	F, 62	8	**0**
H9	Healthy	N/A	F, 57	8	**0**
H10	Healthy	N/A	M, 55	8	**0**

- Cytology Study, Collection System #2 (Comparison with immunofluorescence-based enumeration).

**Patient Info**				**Immuno-Fluorescence (IFC)**				**Cytology**	
**Patient #**	**Type**	**Stage**	**Sex, Age**	**Vol. IFC**	**CTC**	**CTC/mL**	**WBC**	**Vol. PAP**	**# Malignant/mL**
L23	Lung, NSCLC, Squamous	IV	F, 75	6	11	**1.83**	57	6	**0**
L24	Lung, NSCLC, Adeno	IV	M, 39	6	19	**3.17**	46	6	**0.17**

L25	Lung, NSCLC, Adeno	IV	M, 62	6	17	**2.83**	71	6	**0**
L26	Lung, NSCLC, Adeno	IV	M, 52	6	20	**3.33**	87	6	**0.67**
L27	Lung, NSCLC, Adeno	IV	M, 39	6	6	**1.00**	22	6	**1.67**
L28	Lung, NSCLC, Squamous	IV	M, 61	6	33	**5.50**	199	6	**0.17**
L29	Lung, NSCLC, Adeno	IV	M, 82	6	6	**1.00**	102	6	**0.50**
L30	Lung, NSCLC, Adeno	IV	M, 65	6	9	**1.50**	124	6	**0**
L31	Lung, NSCLC, Adeno	IV	F, 58	6	13	**2.17**	114	6	**0.67**
L32	Lung, NSCLC, Adeno	IV	F, 67	6	21	**3.5**	729	6	**0**
B13	Breast, ER-/PR-/HER2-	IV	F, 28	6	67	**11.17**	253	6	**1.17**
B14	Breast ER-/PR-/HER2+	IV	F, 68	6	16	**2.67**	228	6	**0**
B15	Breast, ER+/PR-/HER2+	IV	F, 78	6	20	**3.33**	69	12	**0.33**
B16	Breast, ER-/PR-/HER2-	IV	F, 38	6	34	**5.67**	467	6	**0.17**
B17	Breast, ER-/PR-/HER2+	IV	F, 53	6	25	**4.17**	91	8	**0.50**
B18	Breast, ER+/HER2+	IV	F, 53	6	35	**5.83**	73	8	**0**
B19	Breast, ER+/HER2-	IV	F, 67	6	13	**2.17**	30	8	**0.50**
B20	Breast, ER+/PR+/HER2+	IV	F, 71	6	8	**1.33**	124	6	**0**
B21	Breast, ER+/PR+/HER2 equivocal	IV	F, 38	6	12	**2.00**	189	6	**2.50**
B22	Breast, ER+/HER2+	IV	F, 53	6	7	**1.17**	40	6	**0**
H11	Healthy	N/A	F, 60	6	7	**1.17**	107	6	**0**
H12	Healthy	N/A	F, 52	6	3	**0.50**	62	6	**0**
H13	Healthy	N/A	M 77	6	6	**1.00**	49	6	**0**
H14	Healthy	N/A	M, 36	4	1	**0.25**	12	6	**0**
H15	Healthy	N/A	M, 33	6	4	**0.67**	33	6	**0**

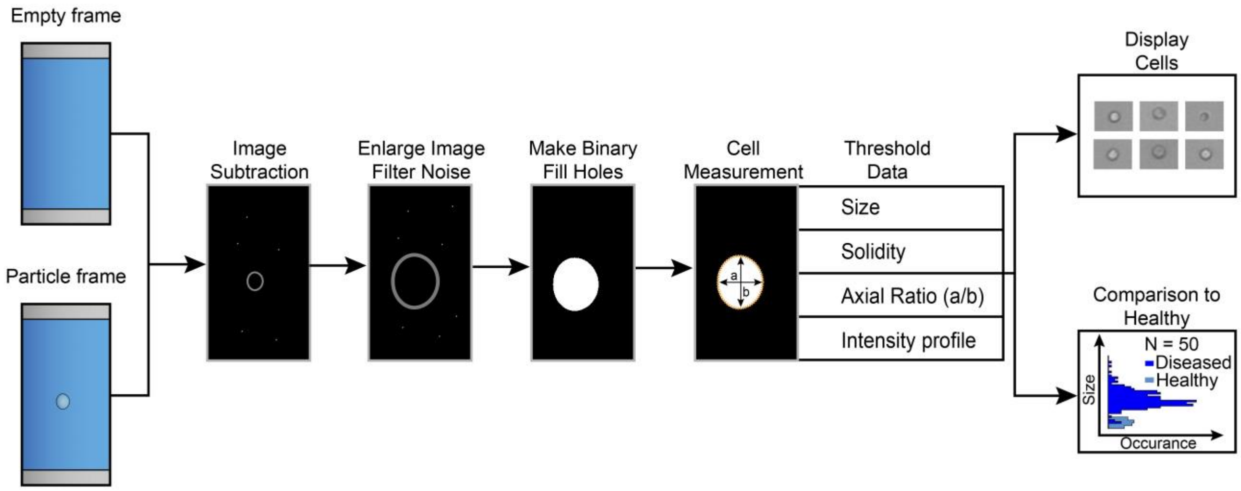

Supp. Figure 1. Custom Image Processing.

A custom image processing algorithm was used to identify cells by their morphology. Objects are detected using a background subtraction method, where the intensity values of an empty frame are subtracted from each ensuing image of the video. Each image is first enlarged by a factor of 3 with interpolation. The images are then converted to binary, and the particle traces are filled with morphological closing (dilation followed by erosion) and measured to determine their diameter, solidity, axial ratios (major axis/minor axis), and cell interior pixel intensity distribution. The data is then thresholded for diameter greater than 12 µm and less than 55 µm. The solidity ranged from 0.7 to 1.0 and the axial ratio ranged from 1 to 1.8. A matrix of cell images and size histograms of cells are shown in the output.

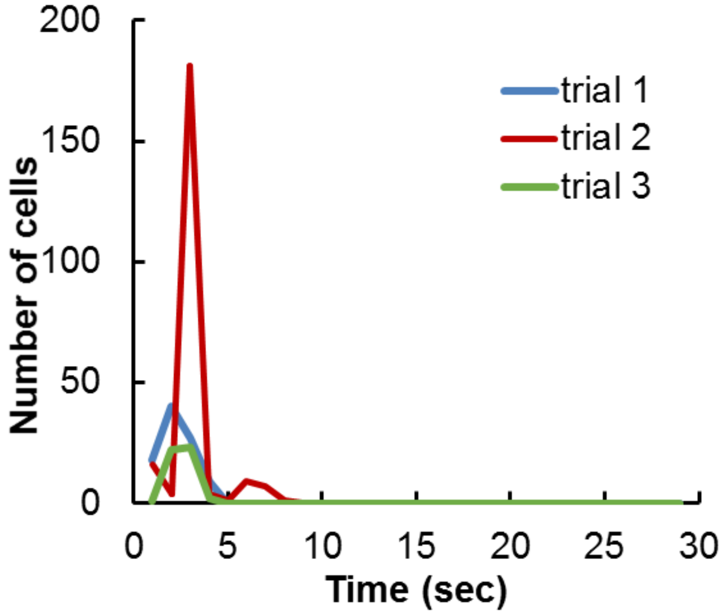

Supp. Figure 2. Release time of cells from vortices. The majority of cells are released from vortices within the first five seconds, while recording is performed over 15 seconds.

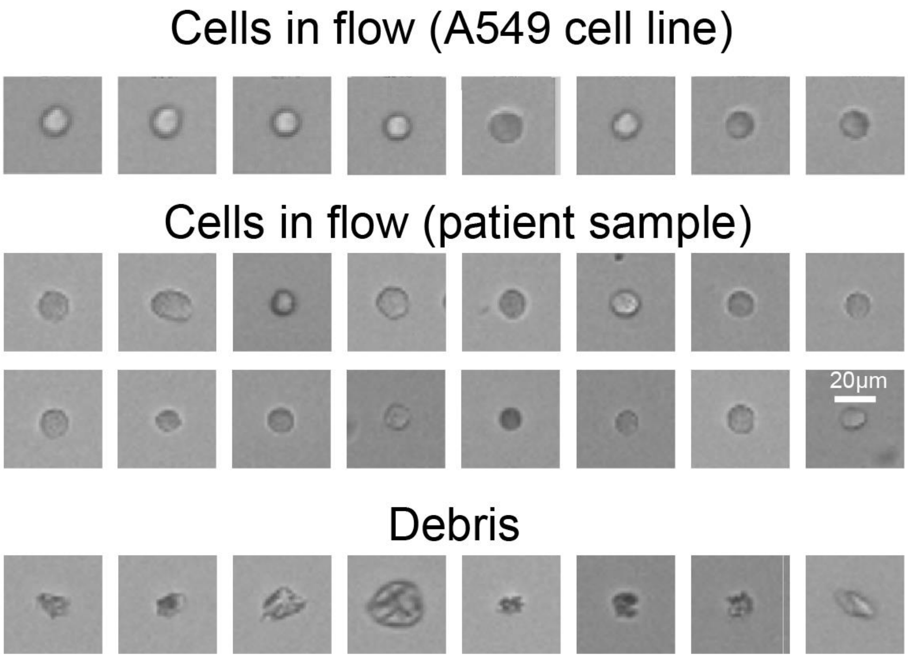

Supp. Figure 3. Gallery of cells in-flow. Cells collected from patient samples were imaged in-flow using bright-field high-speed microscopy. All objects found were thresholded for diameter greater than 12 µm and less than 55 µm. The solidity ranged from 0.7 to 1.0 and the axial ratio ranged from 1 to 1.8. This gallery gives an example of objects that would be classified as cells or as debris respectively.

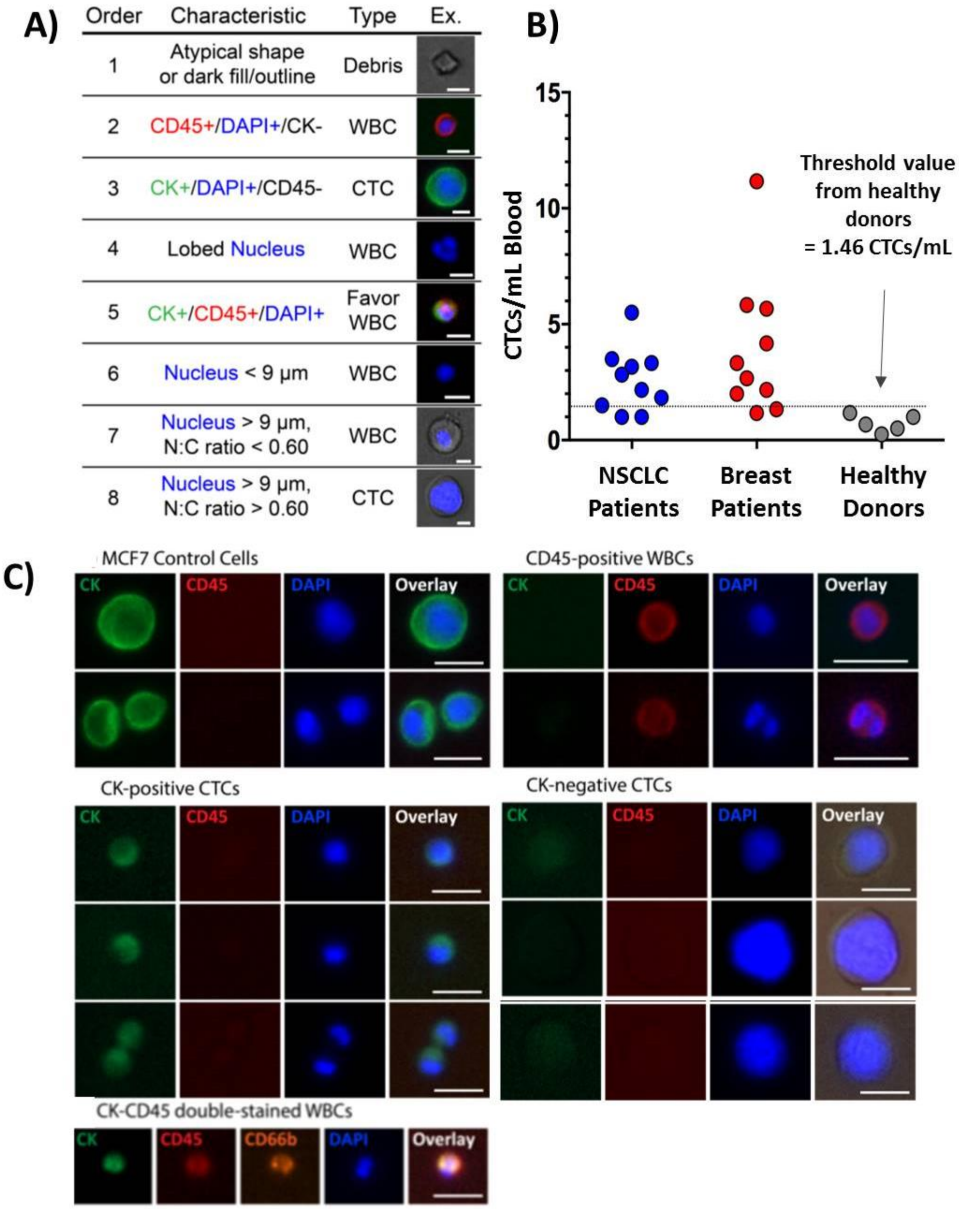

Supp. Figure 4. CTC classification and enumeration by immunofluorescence staining. (A) Cells collected were classified according to their Cytokeratin (CK-FITC, green) and CD45 (PE, red) expression. In addition, nucleated cells were counterstained with DAPI (blue). CTCs were defined as either CK+/CD45-/DAPI+ or DAPI+ only with a large nucleus (>9 µm) and N:C ratio (>0.60). Each cell was compared with the criteria in the order listed until the characteristics matched [Che 2016]. Scale bars represent 10 µm. (B) Using these classification criteria, CTCs were enumerated from 10 lung (NSCLC) and 10 breast cancer patient samples, as well as 5 age-matched healthy donors. A threshold value of 1.46 CTCs/mL was determined from the

enumeration data obtained from the healthy cohort and defined as (mean+2SD). 80% of the lung and 80% of the breast samples had CTCs counts above the threshold value. For NSCLC, 70% of the patients had less than 5 CTCs/mL and 10% had 5-10 CTCs/mL. For breast cancer, 50% of the patients had less than 5 CTCs/mL, 20% had 5-10 CTCs/mL, and 10% had > 10 CTCs/mL. (C) Gallery of cells collected through Vortex HT and stained by immunofluorescence. MCF7 cells, used as a control, strongly stained for CK and were negative for CD45. Most WBCs stained positive for CD45 but negative for CK. Monocytes and lymphocytes were consistently stained strongly whereas granulocytes exhibited weaker CD45 signals. CTCs typically stained weakly or negatively for CK. Several cells are double-stained with both CD45 and CK, but additional staining with CD66b-AF647 confirmed these as activated granulocytes. Large CK-/CD45- cells with high nuclear-to-cytoplasmic ratios were present and defined as CTCs by our criteria. All scale bars represent 20 μm. Figures A and C have been modified from Che et al. 2016 with permission.

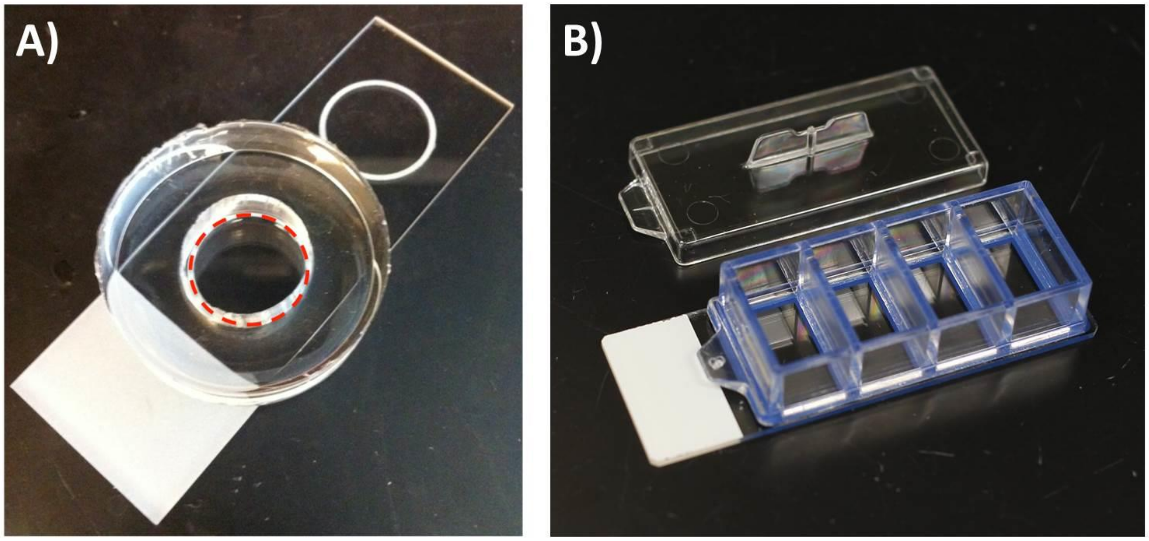

Supp. Figure 5. Collection system for cytology staining. Two collection methods were used to collect the CTCs from the microfluidic device. (A) In the first system, CTCs were collected in a PDMS ring reversibly bonded to a microscope glass slide. (B) The second system consisted of a commercially available chamber glass slide.

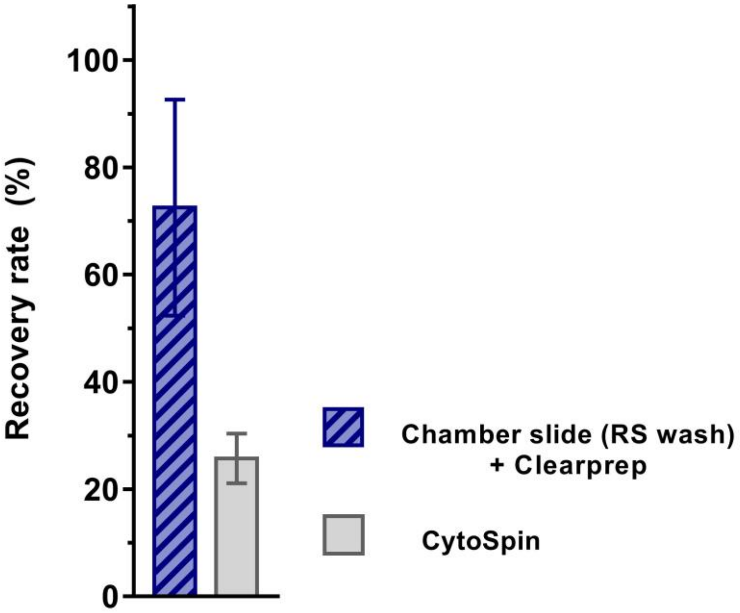

Supp. Figure 6. Comparison of cell recovery rate for chamber slide (+ Clearprep cytology solution) and CytoSpin methods using an MCF7 breast cancer cell line as a model. These 2 methods were compared in parallel, using the same MCF7 cell suspension (N=4). Cell recovery for the chamber slide method was about 70.8% (+/- 16.8%) versus 24.6% (+/- 4.4%) for the CytoSpin.

Supp. Text 1. Papanicolaou staining.

Fixed sample slides were placed into sample holders of the Shandon automated staining machine. The slides were washed in 70% ethanol for 10 sec, rinsed in distilled water for 1 min, then placed in hematoxylin stain for 1.5 min. After, the sample was washed in distilled water for 5 min and then in two 30-sec washes in 95% ethanol. Next, the slide was placed in the OG stain for 3.5 min and then washed in 95% alcohol for 30 sec twice. Lastly, the slides were stained with EA for 6 min. The final washes consisted of two 95% ethanol washes for 30 sec, two 100% ethanol washes for 30 sec, one 100% ethanol washes for 45 sec, two xylene substitute washes for 1 min, and one xylene substitute wash for 2 min. The slides were then taken out of the automated slide stainer and mounted with mounting media and a cover slip.
